# Classification tasks using input driven nonlinear magnetization dynamics in spin Hall oscillator

**DOI:** 10.1038/s41598-023-34849-7

**Published:** 2023-05-16

**Authors:** John Rex Mohan, Arun Jacob Mathew, Kazuma Nishimura, Ruoyan Feng, Rohit Medwal, Surbhi Gupta, Rajdeep Singh Rawat, Yasuhiro Fukuma

**Affiliations:** 1 Department of Physics and Information Technology, Faculty of Computer Science and Systems Engineering, Kyushu Institute of Technology, Iizuka, 820-8502 Japan; 2 Research Center for Neuromorphic AI Hardware, Kyushu Institute of Technology, Kitakyushu, 808-0196 Japan; 3 Natural Sciences and Science Education, National Institute of Education, Nanyang Technological University, Singapore, 637617 Singapore; 4 Present Address: Department of Physics, Indian Institute of Technology, Kanpur, 208016 India; 5 Present Address: Department of Physics, Motilal Nehru National Institute of Technology Allahabad, Prayagraj, 211004 India

**Keywords:** Electronic and spintronic devices, Electrical and electronic engineering

## Abstract

The inherent nonlinear magnetization dynamics in spintronic devices make them suitable candidates for neuromorphic hardware. Among spintronic devices, spin torque oscillators such as spin transfer torque oscillators and spin Hall oscillators have shown the capability to perform recognition tasks. In this paper, with the help of micromagnetic simulations, we model and demonstrate that the magnetization dynamics of a single spin Hall oscillator can be nonlinearly transformed by harnessing input pulse streams and can be utilized for classification tasks. The spin Hall oscillator utilizes the microwave spectral characteristics of its magnetization dynamics for processing a binary data input. The spectral change due to the nonlinear magnetization dynamics assists in real-time feature extraction and classification of 4-binary digit input patterns. The performance was tested for the classification of the standard MNIST handwritten digit data set and achieved an accuracy of 83.1% in a simple linear regression model. Our results suggest that modulating time-driven input data can generate diverse magnetization dynamics in the spin Hall oscillator that can be suitable for temporal or sequential information processing.

## Introduction

Machine learning with artificial neural networks (ANNs) has become an essential component of modern computing^1,2^. ANNs perform classification and pattern recognition tasks by nonlinearly transforming input data into a higher-dimensional space feature map. The ANNs are hierarchically connected units, constructed between an input and an output layer (classifier layer), with a predetermined number of inner layers. The connection strength between each layer, called weights, are trained to perform specific tasks. The inner layers, commonly called “hidden layers”, perform convolution operations in Convolution Neural Networks (CNN) or weighted sum (perceptron) operations in Feed-forward Neural Networks (FNN), followed by a nonlinear activation of inputs^3–5^. Typically, problems with pattern recognition involve Feature Extraction (FE) or Feature Selection (FS) methods for mapping. FE involves the transformation of the original data into feature vectors and their subsequent mapping to a feature space, whereas FS involves choosing the relevant feature among the available states without any functional mapping^3^.

Feature extraction is a computationally intensive process. It requires large matrix operations for weight optimization across multiple connected layers of the network, resulting in a complex learning process and a significant amount of data^6^. The FS method aims to reduce the data storage requirement by removing redundant data and selecting only relevant data via filters, wrappers, and embedded operations^7,8^. However, because of frequent memory access and high memory requirement, traditional von Neumann architecture suffers from speed and efficiency issues. Furthermore, large processing operations hinder the implementation of ANN for real-time processing in memory-constrained devices^4,9^. Neuromorphic computing processors and hardware capable of in-memory computing are being considered for performing various computational tasks^10–15^. Various physical systems including photonics or spintronics based hardware are considered, which can combine processing and memory capabilities to carry out weight optimization, nonlinear activations and in-situ mathematical operations like matrix–vector multiplication^2,15–17^. Despite these advancements, on-device inference and classification of multibit input data on the device itself are scarce^18–20^. By having specialized inference or feature extraction computing units, the energy costs of feature mapping, which make up 90% of the costs in currently used ANNs, can be significantly decreased^15,21^. However, implementing dedicated computing units require overcoming significant hurdles such as, (i) efficient signal processing to generate readily accessible signal parameter identification and computational outputs, (ii) integration with existing complementary metal–oxide–semiconductor (CMOS) circuits, and (iii) adaptability to the existing machine learning algorithms^22^. Therefore, it is important to identify and model physical devices that can perform computational tasks while consuming less power, especially for devices with limited memory.

Spintronic oscillators such as spin-torque oscillators (STOs) and spin Hall oscillators (SHOs) have been widely studied for their use as feature filters in computational tasks such as pattern recognition and classification tasks. In STOs, the spin transfer torque (STT) is induced by the electric current flowing through a multilayer structure consisting of a free magnetic layer and a fixed magnetic layer separated by a non-magnetic conductor or an insulator^23,24^. The electric current passing through the fixed layer is spin-polarized in the direction of magnetization and exerts a torque on the free layer magnetization. The SHO consists of a ferromagnetic (FM) and heavy metal (HM) bilayer structure in which the generation of a pure spin current in the HM via the spin Hall effect (SHE) or the Rashba–Edelstein effect induces the spin orbit torque (SOT)^25–28^. The SHOs have a few additional benefits despite STOs having a higher oscillatory output power. First, they are easier to fabricate due to their simpler bilayer structure^29–32^. Second, the SOT which is caused by the pure spin current from the HM electrode, can be exerted over extended areas in SHOs, whereas the STT in STOs is a localized effect^31,32^. SOT can be successfully used to excite and regulate a variety of magnetization oscillations^33–36^. Furthermore, the development of charge-to-spin conversion efficiency through material engineering and the significant contribution of a field-like term to the SOT, caused by interfacial effects, provides an interesting opportunity to investigate SHO-based neuromorphic hardware^37–40^.

In this article, we use micromagnetic simulations to model a SHO device in the framework of a single domain and take advantage of the microwave spectral properties of its magnetization dynamics to process binary 0 and 1 inputs. We show that the spectral filter technique can be used to directly classify multibit binary data. The present SHO model is a simplified model and based on physically accessible parameters, which makes them easily accessible for feature extraction. The main motivation behind this work is to reduce the dimensionality of feature maps in order to achieve fast and efficient information processing with low training cost. By controlling the magnetization dynamics of the SHO through the parameters of the input pulse pattern, we are able to classify the input sequences that contain 4-binary digit data. Next, we demonstrate recognition of handwritten digits of the Modified National Institute of Standards and Testing (MNIST) handwritten digit database. A SHO device with a modified input pulse pattern requires static readout and training of a linear network to classify handwritten digits.

## Results and discussion

### SHO device model

**Figure 1:**
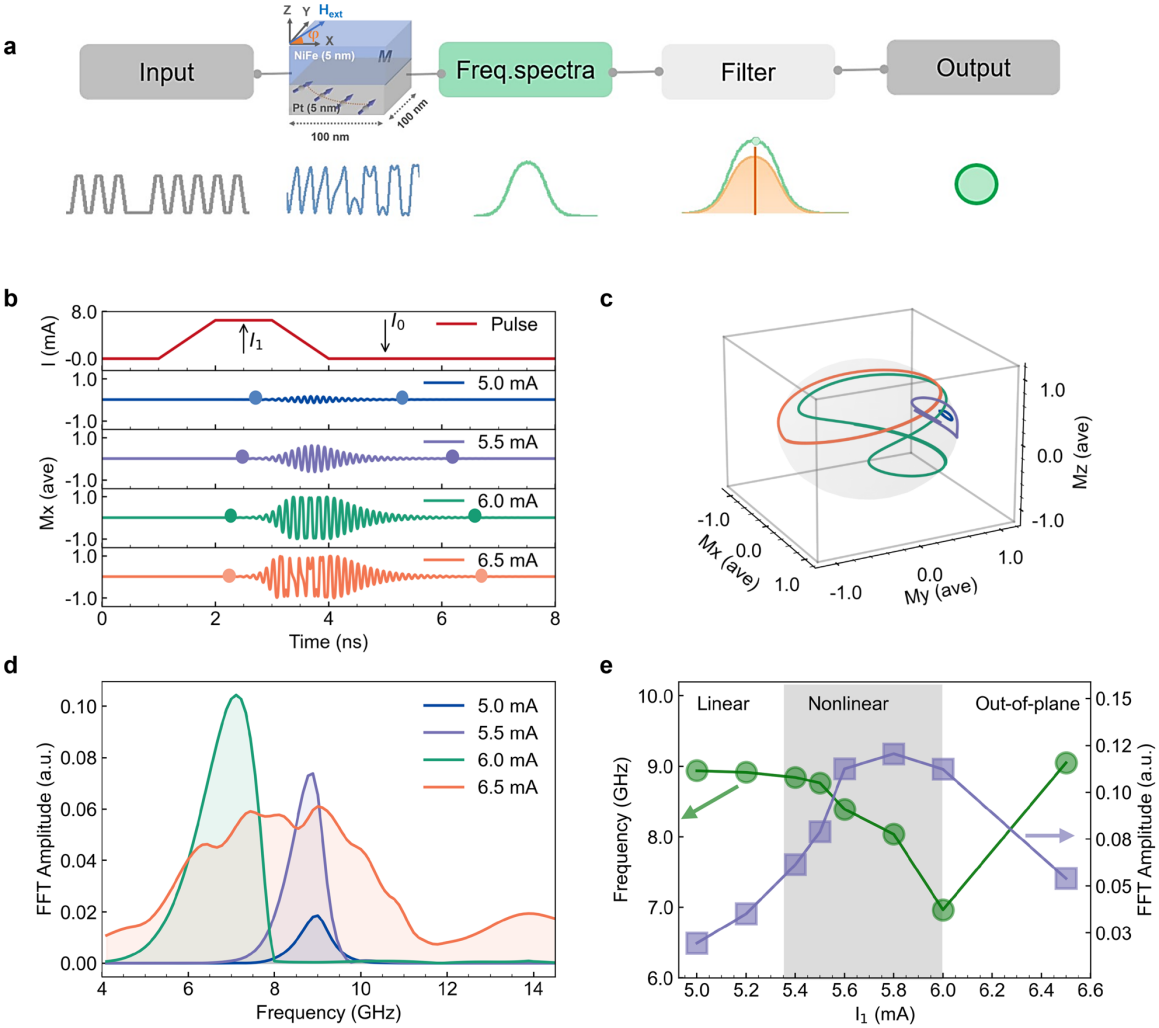
(**a**) Conceptual filtering process for the classification of inputs with use of a spin Hall oscillator. (**b**) Pulsed input current and magnetization dynamics of Mx component for I_1_ = 5.0–6.5 mA, the initiation and relaxation time scales are indicated by colored circles. (**c**) Transition of small angle precession to large angle oscillation trajectories as a function of I_1_. (**d**) Fast Fourier transformed amplitude spectrum of time domain Mx component as a function of I_1_. (**e**) Frequency response and peak amplitude level of FFT spectrum as a function of I_1_.

A conceptual schematization of the SHO-based feature filter is shown in Fig. 1a. It consists of a pulse input, a SHO, a frequency domain output and a filter. The modelled SHO is composed of platinum/permalloy bilayer (Pt/NiFe) with a lateral size of 100 × 100 nm^2^. Each layer has a thickness of 5 nm. The magnetization is aligned at an angle φ = 90° (+ Y), by applying an in-plane magnetic field, ${\upmu }_{0}{\mathrm{H}}_{\mathrm{ext}}=100 {\text{ mT}}$. The SHE in Pt causes spin-dependent electron scattering to the top and bottom surfaces of Pt upon the flow of current ${\mathrm{I}}_{\mathrm{c}}$ in the X direction. This leads to spin accumulation at the interface of Pt/NiFe and a subsequent transfer of spin angular momentum to NiFe results in a transverse flow (Z) of spin current (${\mathrm{I}}_{\mathrm{s}}$). The ratio between charge current density (${\mathrm{j}}_{\mathrm{c}}$) and spin current density (${\mathrm{j}}_{\mathrm{s}}$) is characterized by the spin Hall conversion efficiency, ${\uptheta }_{\mathrm{SH}}$ (spin Hall angle)^25,26^. The ${\mathrm{j}}_{\mathrm{s}}$ results in two SOTs known as field-like torque (FLT) and damping-like torque (DLT). FLT is the result of an interfacial effect due to the strength of spin accumulation at the interface, whereas DLT is the result of bulk phenomena of the SHE^41,42^. Due to the minimal impact of FLT for the 5 nm thick Pt Layer, we have only taken into account the role of DLT on the dynamics of magnetization in the simulations. The natural NiFe damping can be controlled by DLT and fully compensated to produce auto-oscillations in the gigahertz frequency range by increasing ${\mathrm{j}}_{\mathrm{c}}$^29,33,34^. The “Methods” section contains information on the simulation technique and the material parameters. The input data are in the form of current pulses. The binary digits “1” and “0” are encoded as two distinct current values, ${\text{I}}_{1}$ and ${\text{I}}_{0}$, respectively. The output channel is assumed to be of anisotropic magnetoresistance (AMR) based electrical read-out which depends on the magnetization component Mx^24,43^. To represent the collective behavior of the SHO for the given input signal, the fast Fourier transform (FFT) transforms the simulated time-dependent Mx into frequency spectra. The main benefit of frequency domain analysis is the reduced volume of output data for additional computations. The following section will discuss how a filtering mechanism can be used to perform feature extraction and a straightforward linear classification of input data.

### Inherent magnetization dynamics excited by SOT

We begin by examining the magnetization dynamics of the modeled SHO device as a function of ${\mathrm{I}}_{1}$ for a single current pulse with a pulse width ($\uptau$) of 3 ns and pulse rise and fall times of 1 ns. In Fig. 1b, the magnetization component Mx’s temporal evolution is depicted for ${\text{I}}_{1}$ = 5.0, 5.5, 6.0, and 6.5 mA with ${\text{I}}_{0}$ = 0 mA. SOT excitation of a ferromagnetic resonance mode causes magnetization oscillations at ${\text{I}}_{1}$ = 5.0 and 5.5 mA. They exhibit small angle precession, in which the cone angle increases with an increase of ${\text{I}}_{1}$^44–46^. As shown by the colored circles in Fig. 1b, the initiation and relaxation times vary depending on the strength of ${\text{I}}_{1}$. This indicates the manipulation of effective damping by the SOT. These excitations can be converted into self-sustaining auto-oscillations by gradually increasing the precession amplitude with $\uptau$ until it saturates at the limit cycle of auto-oscillation^47^. For ${\text{I}}_{1}$ = 6.0 and 6.5 mA, the oscillations correspond to the in-plane and out-of-plane auto-oscillation modes, respectively. The equilibrium energy, which regulates the auto-oscillation’s limit cycle, is influenced by the device geometry, mode of excitation, and direction of the effective field. The auto-oscillation orbit for the single magnetic domain model is circular in the out-of-plane direction and shaped like a clamshell in the in-plane direction^45^. The single cycle trajectories for each oscillation mode are depicted in Fig. 1c.

In Fig. 1d, the FFT amplitude spectrum for the ${\text{I}}_{1}$ range (5 mA to 6.5 mA) is displayed. For ${\text{I}}_{1}$ < 5.5 mA, the FFT amplitude increases linearly with increasing ${\text{I}}_{1}$, but the oscillation frequency, which can be seen from the peak position of the FFT amplitude in the spectra, is constant at 9.0 GHz. This constant frequency corresponds to the ferromagnetic resonance frequency (Supporting Fig. S1). For 5.5 mA < ${\text{I}}_{1}$ < 6.0 mA, the frequency undergoes a red shift due to the large angle motion of magnetization components, as can be seen in Fig. 1c (${\text{I}}_{1}$ = 6.0 mA), which reduces the effective demagnetization field in the NiFe layer. The magnetization component transverse to ${\mathrm{H}}_{\mathrm{ext}}$ undergoes oscillations at twice the oscillation frequency in order to maintain a constant magnitude and thus reduces the frequency. As ${\text{I}}_{1}$ is increased further, the frequency decreases, reaching 7.1 GHz at ${\text{I}}_{1}$ = 6.0 mA. This frequency shift is attributed to the complex coupling of oscillatory amplitude and phase as predicted by the nonlinear auto-oscillator theory for STOs^48^. For ${\text{I}}_{1}$ = 6.5 mA, the frequency increases to 9.1 GHz and the FFT amplitude reduces due to the out-of-plane oscillation, as can be seen in Fig. 1c (${\text{I}}_{1}$= 6.5 mA). As a result, the magnetization dynamics in the SHO can be divided into three regimes, as shown in Fig. 1e: the linear excitation regime for ${\text{I}}_{1}$ < 5.5 mA, where the frequency is constant with increasing precession amplitudes as a function of ${\text{I}}_{1}$, the nonlinear excitation regime for 5.5 mA < ${\text{I}}_{1}$  < 6.0 mA, where the FFT amplitude is saturated and the frequency decreases drastically with increasing ${\text{I}}_{1}$ and the out-of-plane oscillation regime for ${\text{I}}_{1}$ > 6.5 mA where the frequency increases and the FFT amplitude decreases. These nonlinear frequency amplitude relationships can be used to classify the inputs.

### Binary inputs classification

#### Regular pulse scheme

**Figure 2:**
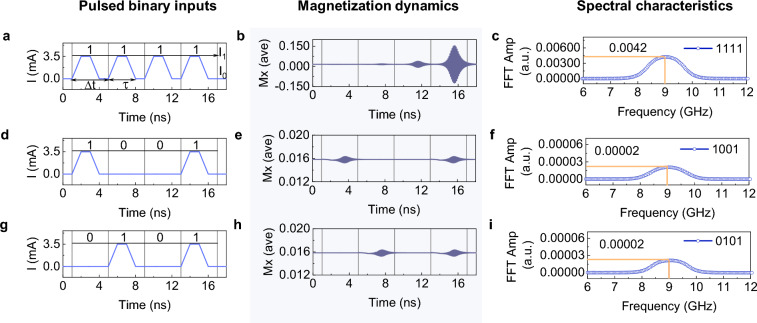
(**a**) Regular pulse scheme for 4-binary digit input pattern 1111 with pulse period (Δt) of 4 ns and pulse width (τ) of 3 ns. (**b**) Magnetization dynamics of Mx component and (**c**) corresponding spectral characteristics. Guide line in FFT shows the amplitude value at 9.0 GHz. Similar input pattern, magnetization dynamics and FFT in (**d–f**) for 1001 pulse pattern, and in (**g–i**) for 0101 pulse pattern.

After investigating magnetization dynamics, we look into the SHO’s ability to classify $\mathrm{n}$-binary input data. The pulse stream is represented by the encoded input signal n – bi(t)$,$ which has current values ${\text{I}}_{0}$ and ${\text{I}}_{1}$ for “1” and “0”, respectively. The pulse period ($\mathrm{\Delta t}$) and width ($\uptau$), respectively, 4 ns and 3 ns. The pulse width $\uptau$, includes a rise time of 1 ns and a fall time of 1 ns. Figure 2 represents 4 – bi(t) input pulse patterns, Mx responses, and FFT amplitude spectra (frequency) with input current values of ${\text{I}}_{1}$ = 3.5 mA and ${\text{I}}_{0}$ = 0 mA, which lies in the linear excitation regime. For the input pattern 1111 in Fig. 2a, the Mx response in Fig. 2b shows the magnetization oscillations with varying amplitude for each “1” input, and the corresponding FFT spectrum is shown in Fig. 2c. For the 1001 pattern in Fig. 2d, the magnetization relaxes to its initial state in the time between the two “1” inputs, as shown in Fig. 2e. The corresponding amplitudes in the FFT spectrum (Fig. 2c,f) for the input patterns of 1111 and 1001 allow one to clearly see the difference in magnetization dynamics.

In order to categorize the patterns of the input data and extract features from the outputs, we employ the classic signal processing tool of filtering. We apply the filter neuron concept, which is inspired by recurrent neural networks’ use of it for feature extraction and handling time-varying outputs, to filter a specific feature in the output data^49,50^. The linear excitation regime frequency, 9.0 GHz, is fixed as the filter and the FFT amplitude value at this frequency  is set as the filter output characteristic feature in order to separate the input patterns in the output spectrum. The filtered amplitude values for the input patterns 1111 and 1001 at 9.0 GHz are 0.0042 and 0.00002, respectively, as shown in Fig. 2c,f. Note that this filtering strategy differs from the standard bit slicing techniques used in the computing paradigm. The bit slicing method maps $\mathrm{n}$ input elements to $\mathrm{n}$ output values and then performs additional computations for weight optimization^9^. But in this case, $\mathrm{n}$ input elements are mapped to a single output value using the quantization technique. This method is well suited for the reduction of output data and does not call for weight optimization for the classification task of input patterns, which can reduce the computation costs^51,52^.

The relaxation of magnetization precession during the interval between two consecutive pulses poses a challenge to the classification task. For the 0101 input pattern in Fig. 2g, the Mx response is displayed in Fig. 2h. Since the magnetization has relaxed to its initial state prior to the second “1” pulse arrival, the individual “1” pulses exhibit the same oscillating amplitude. As can be seen in Fig. 2f,i, the resulting FFT spectra have the same amplitude as the 1001 pattern. In this case, it is not possible to distinguish between the SHO's output and any of the possible 4 – bi(t) input patterns. Input pulse parameters ${\text{I}}_{0}$, ${\text{I}}_{1}$ and $\uptau$ can be varied to affect the dynamics of the magnetization, but in the linear excitation regime, patterns like 1000 and 0001 still produce the same FFT spectra. This is caused by the same magnetization dynamics, but in a different time frame, for each “1” pulse. We refer to this type of input pulse stream as the regular pulse scheme. Supporting Figures S2 and S3 provide an analysis of the magnetization dynamics for various ${\text{I}}_{1}$ values, as well as the resulting FFT spectra and the inability to separate 4 – bi(t) input patterns.

#### Modified pulse scheme

**Figure 3:**
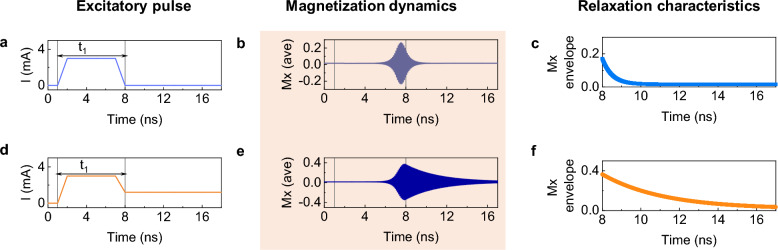
(**a**) Excitatory pulse (I_e_ = 3.0 mA) without offset current (I_0_ = 0 mA). (**b**) Magnetization dynamics of Mx component and (**c**) relaxation characteristics plot from the upper envelope of the Mx time domain data after the pulse is off. (**d**) Excitatory pulse (I_e_) with an offset current value (I_0_ = 1.2 mA). (**e**) Magnetization dynamics of Mx component and (**f**) relaxation characteristics plot of the Mx time domain data.

To tackle the challenge faced in the regular pulse scheme, we resort to modifying the input driven magnetization dynamics rather than the internal structure of the device^53^. Figure 3a depicts the pulse input of an excitatory pulse ${\text{I}}_{\text{e}}$, with a pulse width (${\text{t}}_{1}$) of 7 ns. The Mx oscillation, as shown in Fig. 3b reaches an amplitude of 0.18 and relaxes to the ground state within 2 ns when ${\text{I}}_{\text{e}}$ = 3.5 mA and ${\text{I}}_{0}$ = 0 mA. This can be clearly seen from the upper envelope plot of Mx shown after the pulse is off at 8 ns in Fig. 3c. However, by introducing an offset value for ${\text{I}}_{0}$, the relaxation time can be extended. Figure 3d shows an excitatory pulse with an offset value for ${\text{I}}_{0}$ (${\text{I}}_{\text{e}}$ = 3.5 mA, ${\text{I}}_{0}$ = 1.2 mA). The oscillation amplitude increases to 0.38 due to the increasing precessional amplitude with the offset current, as shown in Fig. 3e, and the relaxation period is extended by 8 ns, as shown in Fig. 3f. This allows us to modify the magnetization dynamics in the SHO during the inputs for different 4 – bi(t) patterns, as will be discussed below.

In order to extend the dynamics of magnetization relaxation for the duration of the input pulse pattern, a modified pulse scheme (${\text{I}}_{\text{mod}}$) that includes the excitatory pulse (${\text{I}}_{\text{e}}$) and a pulse gap ($\updelta$) prior to the introduction of the 4 – bi(t) is used. Hence, the SHO responds to a combination of two input signals, ${\text{I}}_{\text{e}}$ and bi(t) given by,

1 $${\text{I}}_{{{\text{mod}}}} = \left\{ {\begin{array}{*{20}l} {{\text{I}}_{{\text{e}}} ;} & {0 < {\text{t}} < {\text{t}}_{1} } \\ {{\text{n}} - \text{bi}\left( {\text{t}} \right);} & {\text{t} > {\text{t}}_{1} + \updelta } \\ \end{array} } \right..$$

**Figure 4:**
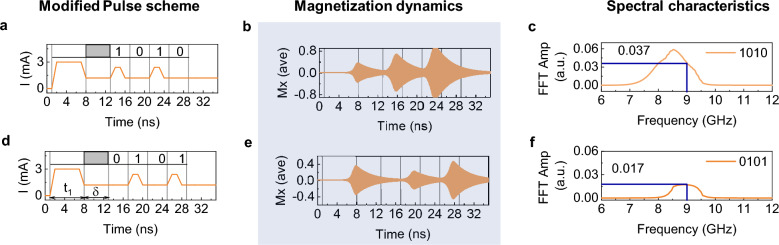
(**a**) Modified pulse scheme with an excitatory pulse I_e_ = 3.0 mA of pulse width t_1_ = 7 ns, for 4-binary digit pattern 1010 with pulse period (Δt) of 4 ns and pulse width (τ) of 3 ns. The pulse gap of δ = 5 ns between I_e_ and 4-binary digit patterns is shown with the grey box. (**b**) Magnetization dynamics of Mx component and (**c**) corresponding spectral characteristic FFT plot. (**d–f**) Similar plots of input pattern, magnetization dynamics and FFT for 0101 pulse pattern. Guide line in FFT plots show the amplitude value at 9.0 GHz.

**Figure 5:**
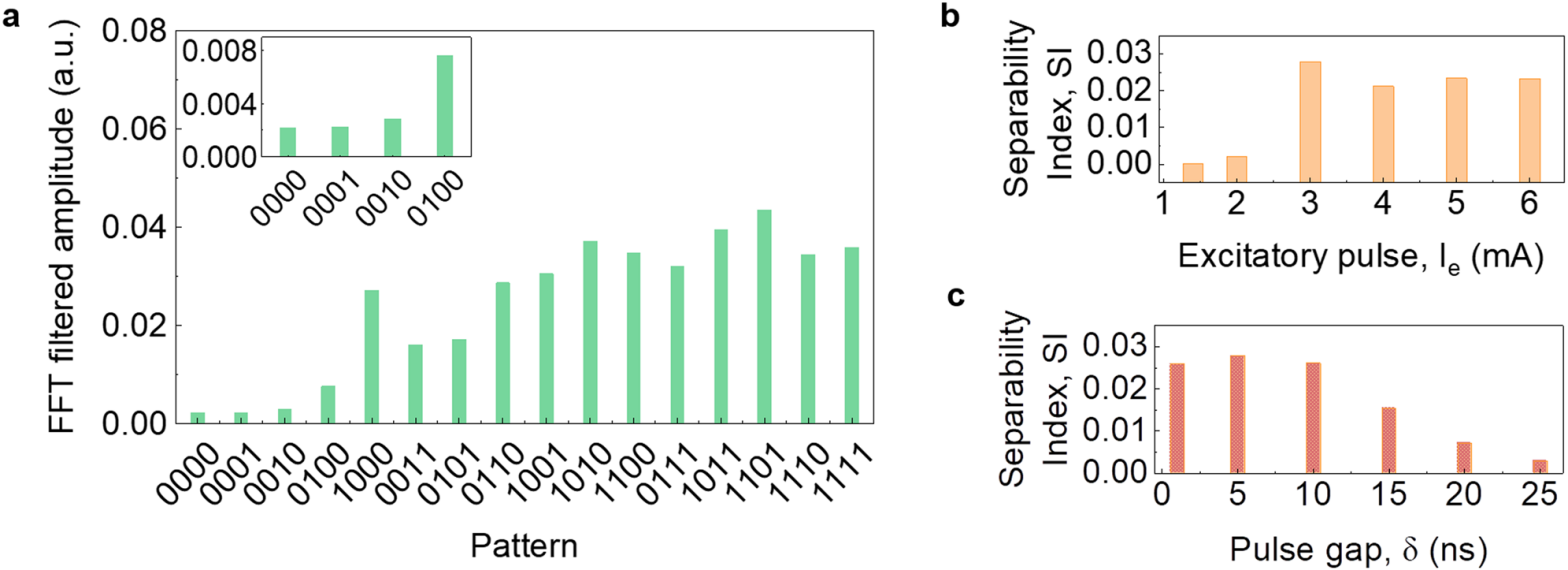
(**a**) Filtered amplitude at frequency of 9.0 GHz for all 16 4-binary digit inputs for the modified pulse scheme with input parameters I_e_ = 3.0 mA, t_1_ = 5 ns, δ = 5 ns, I_0_ = 1.2 mA, I_1_ = 2.4 mA, Δt = 4 ns, τ = 3 ns. (**b**) Separability index for different excitatory pulse current values I_e_ = 1.4–6.0 mA, with δ = 5 ns. (**c**) Separability index for different pulse gap values δ = 1–25 ns with I_e_ = 3.0 mA.

A modified pulse scheme with ${\text{I}}_{\text{e}}$ = 3.0 mA, ${\text{t}}_{1}$ = 7 ns, $\updelta$ = 5 ns, ${\text{I}}_{0}$ = 1.2 mA, ${\text{I}}_{1}$ = 2.4 mA, $\mathrm{\Delta t}$ = 4 ns, $\uptau$ = 3 ns is used for illustration. For simplicity, we denote these input patterns with the above mentioned parameters as ${\mathrm{IP}}_{1}$. Figure 4a shows the input pattern of 1010, the Mx response in Fig. 4b, and the FFT spectrum in Fig. 4c. Similarly, Fig. 4d shows the input pattern for 0101, the Mx response in Fig. 4e, and the FFT spectrum in Fig. 4f. As can be seen from the Mx responses in Fig. 4b,e, each “1” pulse exhibits a different oscillating amplitude as the magnetization dynamics is influenced by both the prior ${\text{I}}_{\text{e}}$ and the corresponding pulses. Since ${\text{I}}_{\text{e}}$’s influence gradually diminishes over time, each output has a unique dynamic, and the degree of influence of previous inputs varies as a function of time. As anticipated, the variation in relaxation dynamics has a significant impact on oscillation amplitude. For the ${\text{I}}_{\text{mod}}$ scheme, the FFTs were obtained from the Mx in the range of input pulse patterns n – bi(t) (Supporting Fig. S4). Figure 4c,f show the variation in the FFT amplitude and frequency as well as the filtered (9.0 GHz) amplitude values 0.037 and 0.017 for the patterns 1010 and 0101, respectively. Because of the input pattern-specific nonlinear magnetization dynamics, ${\text{I}}_{\text{mod}}$ scheme can easily classify these two input patterns from the filtered amplitudes, whereas the previous regular pulse scheme could not. Similarly, the 1000 and 0001 patterns can also be distinguished using the filtered amplitude due to the variations in the relaxation rates. Supporting Figure S5 shows the 16 different n – bi(t) input pattern combinations and their corresponding magnetization dynamics. Figure 5a displays the filtered FFT amplitude for the 16, 4 – bi(t) input patterns using ${\mathrm{IP}}_{1}$. Due to the distinction in the filtered amplitude values, a 4 – bi(t) pattern can be quantized and represented as a 1-dimensional analog output.

#### Seperability index

We further examined how different input ${\text{I}}_{\text{e}}$ and $\updelta$ values in ${\mathrm{IP}}_{1}$ affect the separation of 4-bi(t) digit patterns. The results are compared using a commonly used metric in pattern recognition problems, called separability index, (SI) which is a measure of the average difference between the outputs, for different classes of input parameters^54,55^. SI for ${\mathrm{IP}}_{1}$ with ${\text{I}}_{\text{e}}$ = 1.4–6.0 mA and $\updelta$ = 5 ns is shown in Fig. 5b. For ${\text{I}}_{\text{e}}$ = 1.4 and 2.0 mA, the magnetization precession excited by the first “1” pulse relaxes before the arrival of the next pulse. This results in the FFT amplitudes being very close to each other, leading to a low SI. On the other hand, for ${\text{I}}_{\text{e}}$ = 4.0, 5.0, and 6.0 mA, the magnetization precession excited by the first “1” pulse shows a large amplitude of auto-oscillation, resulting in the consecutive $\mathrm{bi}(\mathrm{t})$ pulses oscillating at the same amplitude. As a result, input patterns like 0111, 1011, 1101, 1110, and 1111 produce FFT amplitudes that are very close to one another. The close range of SI values for ${\text{I}}_{\text{e}}$ = 4.0, 5.0, and 6.0 mA indicates that the excitatory pulse’s ability to distinguish between the various input patterns has been lost. We observed a maximum SI in the case of ${\text{I}}_{\text{e}}$ = 3.0 mA, which indicates that a balance between the ${\text{I}}_{\text{e}}$ pulse and $\mathrm{bi}(\mathrm{t})$ pulses is needed.

Figure 5c shows SI for ${\mathrm{IP}}_{1}$ with a range of pulse gaps, $\updelta$ = 1–25 ns and ${\text{I}}_{\text{e}}$ = 3.0 mA. The effect of ${\text{I}}_{\text{e}}$ on $\mathrm{bi}(\mathrm{t})$ pulses decreases as $\updelta$ increases. Therefore, for input patterns like 0010 and 0001, the FFT amplitudes become similar to one another. ${\text{I}}_{\text{e}}$ has a noticeable impact on $\mathrm{bi}(\mathrm{t})$ for up to 20 ns before it is lost completely at $\updelta$ = 25 ns. For $\updelta$ > 25 ns, the magnetization dynamics are identical to the regular pulse scheme. Therefore, we can conclude that the separation of 4-bi(t) inputs can be optimized to obtain the highest separation at the filtered FFT amplitude with proper ${\text{I}}_{\text{e}}$ and $\updelta$. A comprehensive analysis of magnetization dynamics for various $\updelta$ and ${\text{I}}_{\text{e}}$ are summarized in the Supporting Figs. S6 and S7.

### The MNIST handwritten digit classification

**Figure 6:**
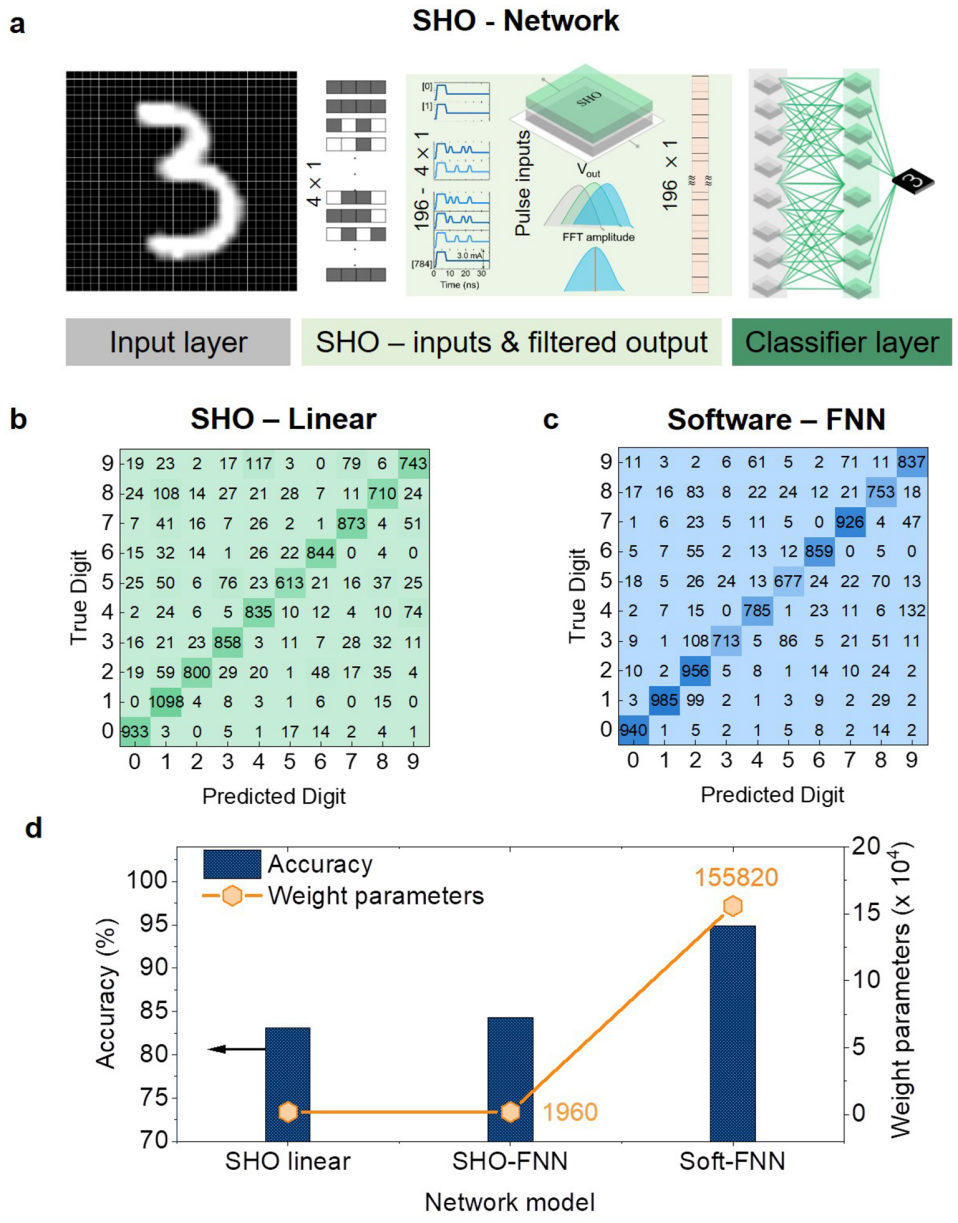
(**a**) Fully connected network model for classification of MNIST handwritten digit datasets with input layer, SHO layer and a classifier layer with 10 neurons for 10 digits. (**b**) Color map shows the confusion matrix classification results for 10,000 test images classified by SHO for a linear regression activation (SHO linear). (**c**) Software implemented feed forward neural network model’s, (software-FNN) classification results color map. (**d**) Comparison of network model implemented with SHO and software as processing unit for filter operations along with classification accuracies (bar graph) and weight parameters (scatter-line plots).

Finally, the SHO device with the modified pulse scheme is evaluated for the classification of handwritten digits of the Modified National Institute of Standards and Technology (MNIST) handwritten database^56^. The database has 60,000 training images and 10,000 test images for the digits 0 to 9. Each image is 28 × 28 pixels in size and is displayed in grayscale, with pixel intensities ranging from 0 to 255. Figure 6a depicts the hypothetical model network, which contains an input layer, the SHO layer, and a classifier layer. The workflow of the network is assumed to have the following layer operations: the images are preprocessed in the input layer so that the original grayscale format is binarized with a threshold (pixel intensity > 125 = 1), where 1 stands for a white pixel and 0 for a black pixel. Following this, the images are divided into 4 pixel segments that move along rows and are then converted into input current pulses, creating a total of 196, $4-\mathrm{bi}(\mathrm{t})$ input patterns. Each of the $4-\mathrm{bi}(\mathrm{t})$ inputs are encoded as a pulse stream and fed to the SHO layer using the modified pulse scheme with ${\mathrm{IP}}_{1}$ parameters. As was discussed in the previous section, the filtered FFT amplitude is collected as the output of the SHO layer for $4-\mathrm{bi}(\mathrm{t})$ pattern at 9.0 GHz. For the results shown here, custom functions were created in Matlab and Python programming languages to replace 4 pixel values with corresponding FFT amplitudes. The classifier layer has 10 nodes that are all fully connected and are used to categorize the 10 handwritten digits from the maximum entry.

The MNIST handwritten digit classification accuracy is evaluated as the ratio of the total number of correctly classified digits to the total number of digits. First, using simple linear regression the weight matrix was calculated using the training data and tested on the test set of 10,000 images. The accuracy obtained by this one-step calculation is 83.1%. The predicted vs. true digit confusion matrix, shown in Fig. 6b as a color map, displays the classification success for the IP_1_ modified pulse scheme. The digit 5 is the least successful at being classified, which lowers the success rate as a whole. In addition, the SHO network model was evaluated in a supervised learning process carried out in Python using the Tensorflow machine learning module^57^. We fixed the softmax activation function for the classifier layer and categorical cross entropy as loss function. The classification accuracy achieved with supervised learning with 20 epochs and 32 batch sizes was 86.6%. This indicates the overall classification accuracy can be improved with classifier layer training. However, our aim is to reduce the previous layer computations for feature extraction.

To illustrate the reduced computations, we performed network training comparing with a software FNN. The software-FNN model consisted of 784 input neurons for each input pixel, a fully connected middle layer of 196 neurons for feature extraction, and ten neurons (the classifier layer) for classification. We used the rectified linear activation function (ReLU) for the feature extraction layers and the softmax activation function for the classifier layer. It is clear that the software-FNN requires 196 neurons and associated computations to generate 196 features for the classifier layer, similar to the SHO layer outputs. The confusion matrix of the software-FNN, shown in Fig. 6c, achieved an accuracy of 93.1%. When compared to the SHO network, the software-FNN improves accuracy by 7.5%. However, the computational cost of calculating the weight matrix is high. Figure 6d shows the accuracy and weight parameters required for the classification for the linear SHO (single linear weight computation), SHO-FNN (supervised learning), and software-FNN. It can be seen that with the use of the SHO layer, the weight computations required for a software network can be significantly reduced from 155,820 weight parameters to 1960 parameters. The ability of the SHO layer to directly infer distinct outputs for $4-\mathrm{bi}(\mathrm{t})$ patterns reduces the computation required. Although there is a trade-off between recognition accuracy and inference computations for the MNIST handwritten digit classification due to various factors like binarization of images, choice of classifier layer training methods, and loss functions, the SHO network is still favorable since it can achieve 83.1% with linear regression. Moreover, a sequence of 4 binary digits can be easily classified without any requirement of weight storage or computations, which can be beneficial for applications that need faster inference with reduced computations. Nevertheless, there is a huge opportunity to engage in the co-design of SHO as a dedicated feature mapping layer alongside already existing hardware and algorithms.

## Conclusion

In this study, a single spin Hall oscillator capable of real-time classification of sixteen different binary $4-\mathrm{bi}(\mathrm{t})$ patterns (0000 to 1111) was demonstrated. Control and tuning of the intrinsic magnetization oscillations of the spin Hall oscillator were performed by modifying an input digit pulse pattern. The performance of the model was tested with the standard MNIST handwritten digit data set classification and achieved an accuracy of 83.1% with a linear training network and reduced output layer computations. Hence, these results are expected to provide an insight into the controlling and tuning of spintronic oscillators for real time and on-device neuromorphic computations.

## Methods

### Micromagnetic simulations

The micromagnetic simulations were carried out using the LLG micromagnetic simulator in the framework of a single domain model^58–60^. The temporal dynamics of the ferromagnetic layer was solved by the Landau-Lifshitz-Gilbert (LLG) equation with the spin transfer torque term,

2 $$\frac{\mathrm{d}\widehat{\mathbf{m}}}{\mathrm{dt}}=-\upgamma \widehat{\mathbf{m}}\times {\upmu }_{0}{\mathbf{H}}_{\mathrm{eff}}+\mathrm{\alpha }\widehat{\mathbf{m}}\times \frac{\mathrm{d}\widehat{\mathbf{m}}}{\mathrm{dt}}-\upgamma \frac{\mathrm{\hslash }}{2\left|\mathrm{e}\right|}\frac{{\uptheta }_{\mathrm{SH}} \left|{\mathrm{j}}_{\mathrm{c}}\right|}{{\mathrm{M}}_{\mathrm{s}}{\mathrm{t}}_{\mathrm{FM}}}\left(\widehat{\mathbf{m}}\times \left(\widehat{\mathbf{m}}\times \widehat{{\varvec{\upsigma}}}\right)\right),$$

where $\widehat{\mathbf{m}}=\frac{\mathbf{M}}{{\mathrm{M}}_{\mathrm{s}}}$ is the normalized magnetization vector, γ is the gyromagnetic ratio, α is the Gilbert damping parameter, ${\upmu }_{0}$ is the vacuum permeability, ${\mathrm{M}}_{\mathrm{s}}$ is the saturation magnetization, $\mathrm{\hslash }$ is the reduced Planck constant, $e$ is the electron charge, and ${\mathrm{t}}_{\mathrm{FM}}$ is the thickness of the magnetic layer. The effective field ${\mathbf{H}}_{\mathrm{eff}}$ includes the external magnetic field, the magneto-crystalline anisotropy field, and the demagnetization field. ${\uptheta }_{\mathrm{SH}}$ is the spin Hall angle, which characterizes the conversion efficiency of charge current density ${\widehat{\mathbf{j}}}_{\mathbf{c}}$ to spin current density ${\widehat{\mathbf{j}}}_{\mathbf{s}}$ in the heavy metal layer. $\widehat{{\varvec{\upsigma}}}=-\mathrm{sgn}{\uptheta }_{\mathrm{SH}}(\widehat{\mathbf{z}}\times {\widehat{\mathbf{j}}}_{\mathbf{c}})$ is the orientation of spin injected into the ferromagnet, where $\widehat{\mathbf{z}}$ and ${\widehat{\mathbf{j}}}_{\mathbf{c}}$ are the unit vectors in the direction of surface normal and the electrical current, respectively. The $-\mathrm{sgn}$ factor changes with the position of ferromagnet, i.e., if the ferromagnet is atop the HM, $\widehat{\mathbf{z}}$ would face into the HM, or if the ferromagnet is below the HM, $\widehat{\mathbf{z}}$ would face into the ferromagnet but it’s sign would be opposite to the prior case. In accordance with experiments, the material parameters used in the simulations^38,39^, are: ${\upmu }_{0}{\mathrm{M}}_{\mathrm{s}}=1.0 {\text{T}}$, $\mathrm{\alpha }=0.02$, ${\uptheta }_{\mathrm{SH}}=0.07$, resistivities ${\uprho }_{\mathrm{NiFe}}=4.5 \times {10}^{-7}\mathrm{ \Omega m}$ and ${\uprho }_{\mathrm{Pt}}=2.0 \times {10}^{-7}\mathrm{ \Omega m}$. The magnetic anisotropy is ignored, and an in-plane external magnetic field of strength ${\upmu }_{0}{\mathrm{H}}_{\mathrm{ext}}=100 {\text{mT}}$ is applied to saturate the magnetization along the + Y direction.

### Seperability index

The separability index (SI) were calculated between the $\mathrm{N}$ different outputs (${\mathrm{a}}_{\mathrm{i}},..,{\mathrm{a}}_{\mathrm{n}}$) of the spin Hall oscillator (filtered FFT amplitudes), corresponding to each of the $\mathrm{N}$ different input patterns via,

3 $$\mathrm{SI}= \sum_{\mathrm{i}=1}^{\mathrm{N}}\sum_{\mathrm{j}=1}^{\mathrm{N}}\frac{{\mathrm{a}}_{\mathrm{i}}-{\mathrm{a}}_{\mathrm{j}}}{{\mathrm{N}}^{2}},$$

where $\mathrm{i}$ and $\mathrm{j}$ are the indices of summation and $\mathrm{N}=16$ (16 patterns). The SI value of a particular input current scheme indicates how well the different input patterns can be distinguished from the closeness of the output values. Thus, we can infer that the input current scheme having the highest SI value is the most suited for performing classification and recognition tasks.

### Network training and testing

#### Linear regression

For the SHO based network training, all of the images in the MNIST dataset were binarized first, and with custom functions another dataset replacing the 4 × 1 or 2 × 2 pixels with FFT amplitudes was created. For the direct software based networks implementation, we used the inbuilt Matlab/Python functions for converting black and white images within the respective classification programs. For the linear regression readout layer, we used linear regression function executed in Matlab software. During the learning process, for a particular image, the output amplitudes were mapped into a one-dimensional feature vector of 196 elements. This process was repeated for all 60,000 training images, creating a 60,000 × 196 output matrix **O**. Then, a 196 × 10 weight matrix **W** (where each column corresponds to each digit from 0 to 9) was calculated using the output matrix **O** and a label matrix **L** (60,000 × 10) containing the true labels for each training image. In each row of the label matrix, the ($\mathrm{l}+1$)th column had a value of 1 and the remaining columns had a value of 0. $\mathrm{l}$ is the true digit ($\mathrm{l}=0, 1...,9$).

In linear regression, assuming a linear relationship between the output matrix **O** and the label matrix **L**,

4 $$\mathbf{O}\mathbf{W}=\mathbf{L},$$

the weight matrix **W** was calculated using pseudo inverse **W**,

5 $${\mathbf{W}} = {\mathbf{O}}^{\dag } {\mathbf{L}}.$$

#### Nonlinear activation functions

The SHO and software-FNN network models were evaluated in a supervised learning process performed in Python using the Tensorflow. All the networks were trained for 20 epochs for the weight optimization. The categorical cross-entropy error is reduced using the ADAM optimizer with default learning rate and a batch size of 32. The activation functions ReLU and softmax for the input (z) are given by,

6 $$\mathrm{ReLU}\left(\mathrm{z}\right)=\mathrm{max}\left(0,\mathrm{z}\right),$$

7 $$\mathrm{softmax}\left({\mathrm{z}}_{\mathrm{i}}\right)=\frac{{\mathrm{e}}^{{\mathrm{z}}_{\mathrm{i}}}}{{\sum }_{\mathrm{j}=1}^{\mathrm{K}}{\mathrm{e}}^{{\mathrm{z}}_{\mathrm{j}}}}.$$

The softmax ${\mathrm{z}}_{\mathrm{i}}$ are the results of previous layer and $\mathrm{K}$ is the number of outputs, which corresponds to the number of classes to be distinguished ($\mathrm{K}=10$ for MNIST handwritten digits 0–9).

## Supplementary Information

Supplementary Information.

## Data Availability

The datasets generated and analysed for this study is available from Kyushu Institute of Technology repository at http://hdl.handle.net/10228/00008940. The MNIST dataset is available at http://yann.lecun.com/exdb/mnist.
