# Supplementary material for: Classification tasks using input driven nonlinear magnetization dynamics in spin Hall oscillator
Source: Sci Rep. 2023 May 16;13:7909. doi: 10.1038/s41598-023-34849-7 (PMC10188540; doi:10.1038/s41598-023-34849-7)
Supplement: Supplementary file 1 — Supplementary Information. [file 41598_2023_34849_MOESM1_ESM.pdf]

Classification tasks using input driven nonlinear magnetization dynamics in spin Hall oscillator

John Rex Mohan[1], Arun Jacob Mathew[1], Kazuma Nishimura[1], Ruoyan Feng[1], Rohit Medwal[3], Surbhi Gupta[3], Rajdeep Singh Rawat[3] and Yasuhiro Fukuma[1,2*]

[1]Department of Physics and Information Technology, Faculty of Computer Science and Systems Engineering, Kyushu Institute of Technology, Iizuka 820-8502, Japan
[2]Research Center for Neuromorphic AI hardware, Kyushu Institute of Technology, Kitakyushu 808-0196, Japan
[3]Natural Sciences and Science Education, National Institute of Education, Nanyang Technological University, Sinagpore, 637617, Singapore

*e-mail: fukuma@phys.kyutech.ac.jp

Supporting Materials

Supplementary material 1
Ferromagnetic resonance.

Supplementary material 2
Investigation of magnetization dynamics and 4-bit digit pattern separation on regular pulse scheme with $I_0 = 0$, $I_1 = 4.0$ & 6.0 mA, $\Delta t = 4$ ns, $\tau = 3$ ns.

Supplementary material 3
Investigation of magnetization dynamics and 4-bit digit pattern separation on regular pulse scheme with $I_0 = 0$ mA, $I_1 = 3.5$ mA, $\Delta t = 4$ ns, $\tau = 1.5$ ns & 3.6 ns.

Supplementary material 4
Magnetization dynamics of 16 combinations of 4-bit digit pattern separation on modified pulse scheme with $I_e = 3.0$ mA, $\delta = 5$ ns, $I_0 = 1.2$ mA, $I_1 = 2.4$ mA, $\Delta t = 4$ ns, $\tau = 3.0$ ns.

Supplementary material 5
Investigation of magnetization dynamics and 4-bit digit pattern separation on modified pulse scheme with I_e =1.4 mA, δ =5 ns, I_0 =1.2 mA, I_1 =2.4 mA, Δt =4 ns, τ =3ns.

Supplementary material 6
Investigation of magnetization dynamics and 4-bit digit pattern separation on modified pulse scheme with I_e =5.0 mA, δ =5 ns, I_0 =1.2 mA, I_1 =2.4 mA, Δt =4 ns, τ =3ns.

Supplementary material 7
Investigation of magnetization dynamics and 4-bit digit pattern separation on modified pulse scheme with I_e =5.0 mA, δ =1, 20 & 25 ns, I_0 =1.2 mA, I_1 =2.4 mA, Δt =4 ns, τ =3ns.

Supplementary material 8. Classification of inputs in the presence of thermal effects.

Supplementary material 1: Ferromagnetic resonance.

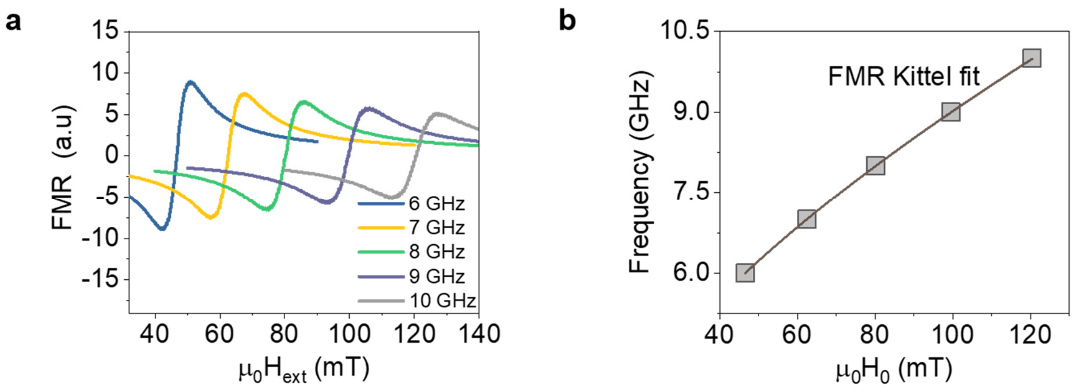

Figure S1. a. Simulated ferromagnetic resonance (FMR) for modelled spin Hall oscillator (SHO) in the main text. The applied magnetic field (H_{ext}) is oriented along Y axis and the oscillating magnetic field (H_{rf}) at a fixed microwave frequency is applied along X axis with a strength of 1 mT.The H_{ext} is sweeped for fixed frequency. **b.** The obtained resonant field (H_o) as a function of the applied frequency. The data are fitted with the Kittel equation $f = \mu_0\gamma/2\pi\sqrt{H_o(H_o + M_{eff})}$ yielding an effective magnetization, $\mu_0 M_{eff} = 1.0$ T with gyromagnetic ratio $\gamma/2\pi = 27.3$ GHz/T.

Supplementary material 2: Investigation of magnetization dynamics and 4-bit digit pattern separation on regular pulse scheme with $I_0 = 0$, $I_1 = 4.0$ & 6.0 mA, $\Delta t = 4$ ns, $\tau = 3$ ns.

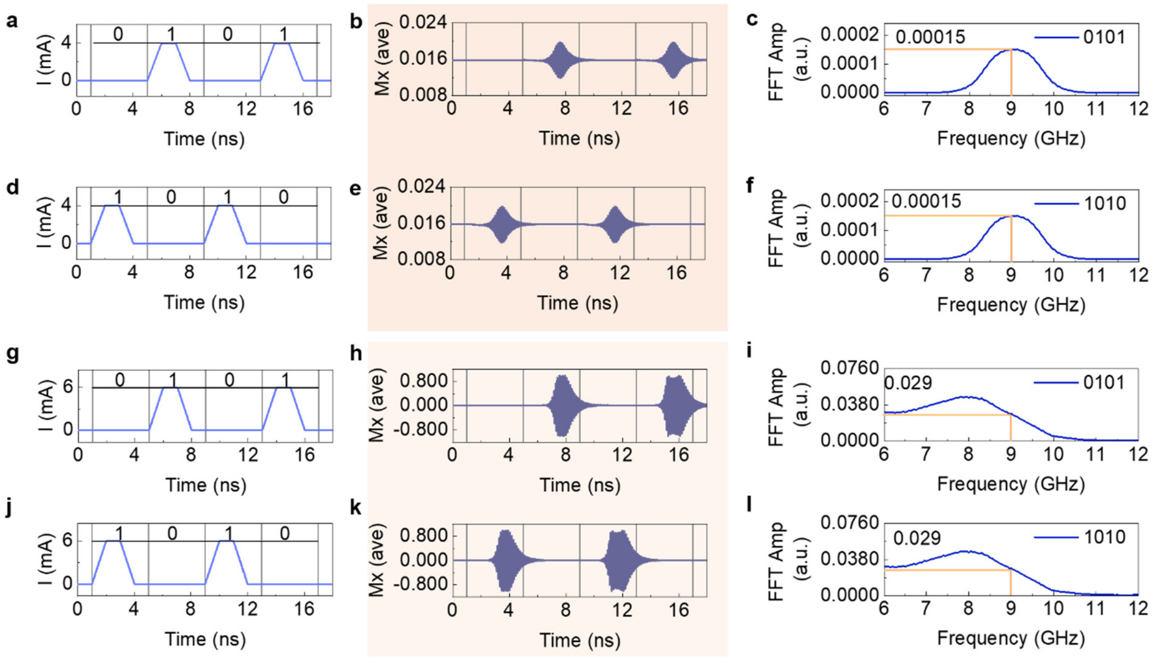

Figure S2. Figures **a** and **d** show the 4-bit binary input pulse patterns with input parameters $I_0 = 0$, $I_1 = 4.0$ mA, $\Delta t = 4$ ns and $\tau = 3$ ns, for the 4-bit patterns 0101 and 1010 respectively. For both pattern 0101 and pattern 1010, the Mx oscillation amplitudes in the Figs. **b** and **e** corresponding to the input bit 1 are the same. This prevents the separation of the two patterns as can be seen from the similar value of FFT amplitude in Figs **c** and **f**. Figures **g** and **j** show the 4-bit binary input pulse with input parameters with I_1 in the nonlinear regime $I_0 = 0$, $I_1 = 6.0$ mA, $\Delta t = 4$ ns and $\tau = 3$ ns, for the 4-bit patterns 0101 and 1010 respectively. For both pattern 0101 and pattern 1010, the Mx responses are auto-oscillations as shown in the Figures **h** and **k,** where bit 1 pulses oscillate at the same amplitude level. This again prevents the separation of the two patterns as seen from the similar value of FFT amplitude at the filtering frequency of 9.0 GHz as shown the Figs **i** and **l**.

Supplementary material 3: Investigation of magnetization dynamics and 4-bit digit pattern separation on modified pulse scheme with $I_0 = 0$ mA, $I_1 = 3.5$ mA, $\Delta t = 4$ ns, $\tau = 1.5$ ns & 3.6 ns.

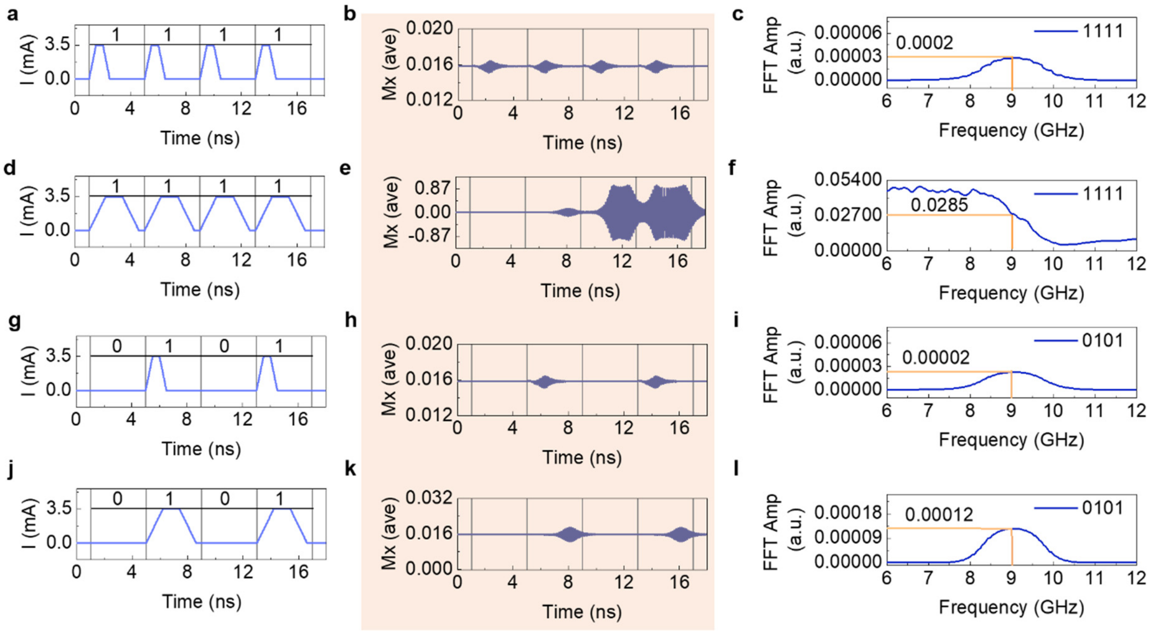

Figure S3. Figure. **a**(**d**) shows the 4-bit binary input pulse with input parameters $I_0 = 0$, $I_1 = 3.5$ mA, $\Delta t = 4$ ns and $\tau = 1.5$ ns (3.6 ns for **d**), for the 4-bit pattern 1111. For $\tau = 1.5$ ns (Fig. **b**), due to the relaxation of the excited small angle precession, Mx amplitudes corresponding to each of input bit 1 pulses are the same. However, for $\tau = 3.6$ ns (Fig. **e**), the next bit 1 pulse arrives before the relaxation of the previously excited Mx precession, leading to progressively increasing amplitudes of oscillation. Figures **c** and **f** give the FFT amplitude spectra for 4-bit pattern 1111 for $\tau = 1.5$ ns & 3.6 ns respectively. Figure **g** (**j**) shows the 4-bit binary input pulse with input parameters $I_0 = 0$, $I_1 = 3.5$ mA, $\Delta t = 4$ ns and $\tau = 1.5$ ns (3.6ns), for the 4-bit pattern 0101. For both $\tau = 1.5$ ns (Fig. **h**) and $\tau = 3.6$ ns (Fig. **k**), the Mx amplitudes corresponding to both the bit 1 pulses are the same. Figures **i** and **l** give the FFT amplitude spectra for 4-bit pattern 1111 for $\tau = 1.5$ ns & 3.6 ns respectively. Similar to Supporting information section S3, this prevents the separation of any cyclic permutations of 4-bit patterns for both $\tau = 1.5$ ns and $\tau = 3.6$ ns.

Supplementary material 4: Magnetization dynamics of 16 combinations of 4-bit digit pattern separation on modified pulse scheme with $I_e = 3.0$ mA, $\delta = 5$ ns, $I_0 = 1.2$ mA, $I_1 = 2.4$ mA, $\Delta t = 4$ ns, $\tau = 3.0$ ns.

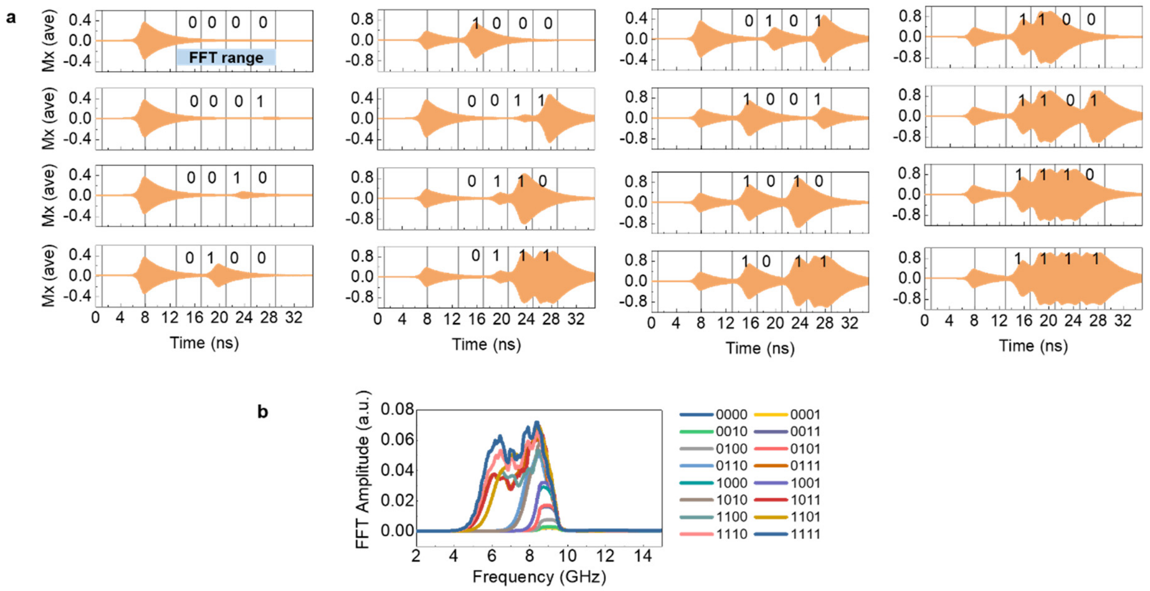

Figure. S4. Figure **a** shows the magnetization dynamics corresponding to the 16 different 4-bit input patterns for the modified pulse scheme with input parameters $I_e = 3.0$ mA, $\delta = 5$ ns, $I_0 = 1.2$ mA, $I_1 = 2.4$ mA, $\Delta t = 4$ ns, $\tau = 3.0$ ns. Figure **b** gives the FFT amplitude spectra corresponding to the 16 different 4-bit input patterns. Note that the FFTs are calculated using Mx in the range of the input patterns as shown in the 0000 bit pattern.

Supplementary material 5: Investigation of magnetization dynamics and 4-bit digit pattern separation on modified pulse scheme with I_e =1.4 mA, δ =5 ns, I_0 =1.2 mA, I_1 =2.4 mA, Δt =4 ns, τ =3ns.

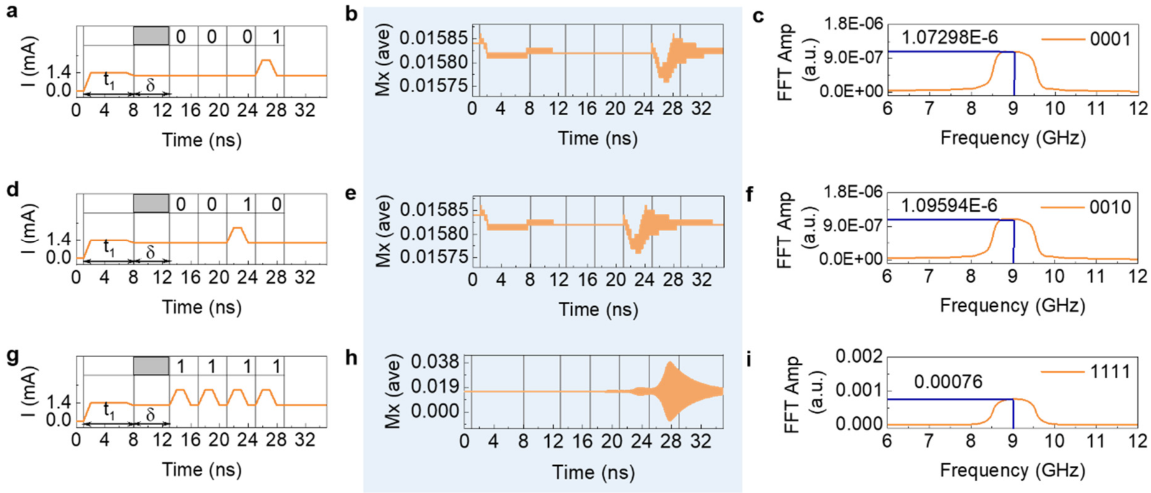

Figure S5. Figures **a**, **b** and **c** show the 4-bit binary input pulse, corresponding magnetization dynamics and FFT amplitude spectra respectively for the 4-bit pattern 0001 with input parameters I_e =1.4 mA, δ =5 ns, I_0 =1.2 mA, I_1 =2.4 mA, Δt =4 ns, τ =3ns. Similarly, Figs. **d** (**g**), **e** (**h**) and **f** (**i**) show the 4-bit binary input, pulse corresponding magnetization dynamics and FFT amplitude spectra respectively for the 4-bit pattern 0010 (1111) with the same input parameters. Note the difference betweeen the FFT amplitude values filtered at 9.0 GHz. The low value of separability index (SI) reported for I_e =1.4 mA can be directly correlated with filtered FFT amplitude values being in close range for the different 4-bit input patterns.

Supplementary material 6: Investigation of magnetization dynamics and 4-bit digit pattern separation on modified pulse scheme with I_e =5.0 mA, δ =5 ns, I_0 =1.2 mA, I_1 =2.4 mA, Δt =4 ns, τ =3ns.

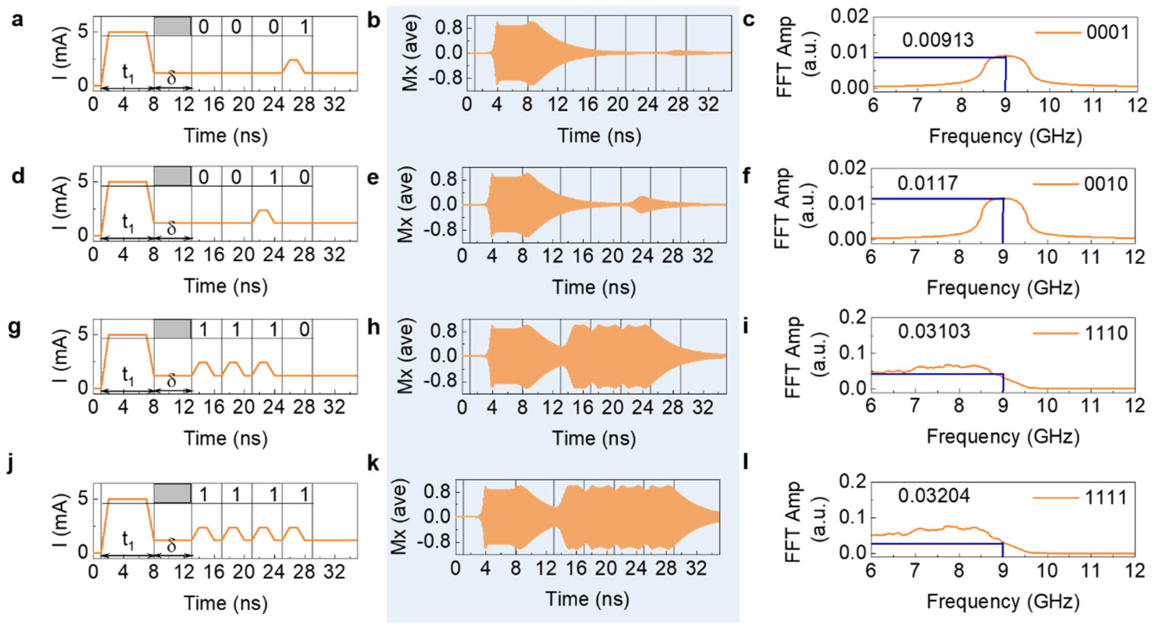

Figure. S6. Figures **a**(**d**), **b**(**e**) and **c**(**f**) show the 4-bit binary input pulse, corresponding magnetization dynamics and FFT amplitude spectra respectively for the 4-bit pattern 0001 (0010) with input parameters I_e =5.0 mA, δ =5 ns, I_0 =1.2 mA, I_1 =2.4 mA, Δt =4 ns, τ =3ns. Similarly, Figures **g**(**j**), **h**(**k**) and **i**(**l**) show the 4-bit binary input pulse, corresponding magnetization dynamics and FFT amplitude spectra respectively for the 4-bit pattern 1110(1111) with the same input parameters. Note the difference betweeen the FFT amplitude values filtered at 9.0 GHz. The high value of separability index(SI) reported for I_e =5.0 mA can be directly correlated with the filtered FFT amplitude values for the different 4-bit input patterns. However, the SI for I_e =5.0 mA is lower than that of I_e =3.0 mA because, as seen in Figs. **h** & **k**, for I_e =5.0 mA, the precessions excited by the excitatory pulse are already in the auto-oscillation mode, leading to saturated Mx amplitudes for all subsequent input bit 1 pulses. This leads to the filtered FFT amplitude values being much closer for I_e =5.0 mA, causing slightly lower SI as compared to I_e =3.0 mA.

Supplementary material 7: Investigation of magnetization dynamics and 4-bit digit pattern separation on modified pulse scheme with I_e =5.0 mA, δ =1, 20 & 25 ns, I_0 =1.2 mA, I_1 =2.4 mA, Δt =4 ns, τ =3ns.

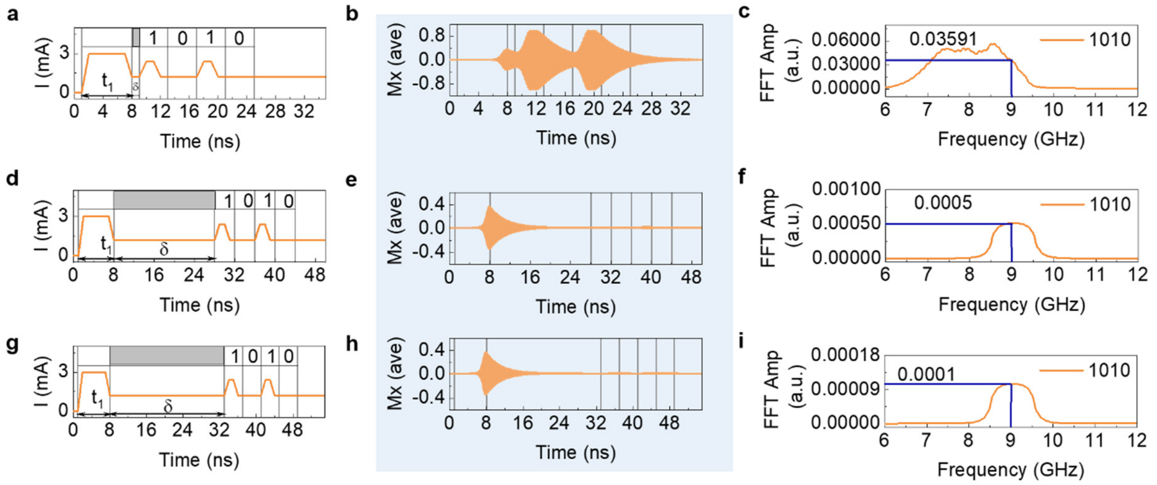

Figure. S7. Figures **a**(**d**,**g**), **b**(**e**,**h**) and **c**(**f**,**i**) show the 4-bit binary input pulse, corresponding magnetization dynamics and FFT amplitude spectra respectively for the 4-bit pattern 1010 with input parameters I_e =5.0 mA, δ = 1 ns (20 ns, 25 ns), I_0 =1.2 mA, I_1 =2.4 mA, Δt =4 ns, τ =3ns. The effect of the excitatory pulse on the input binary pattern decreases with increasing δ. This leads to decreasing Mx amplitudes and therefore decreasing filtered FFT amplitudes at 9.0 GHz with increasing δ. Moreover, the reduced filtered FFT amplitudes for all 16 combinations of 4-bit input patterns causes the SI to decrease with increasing δ.

Supplementary material 8. Classification of inputs in the presence of thermal effects.

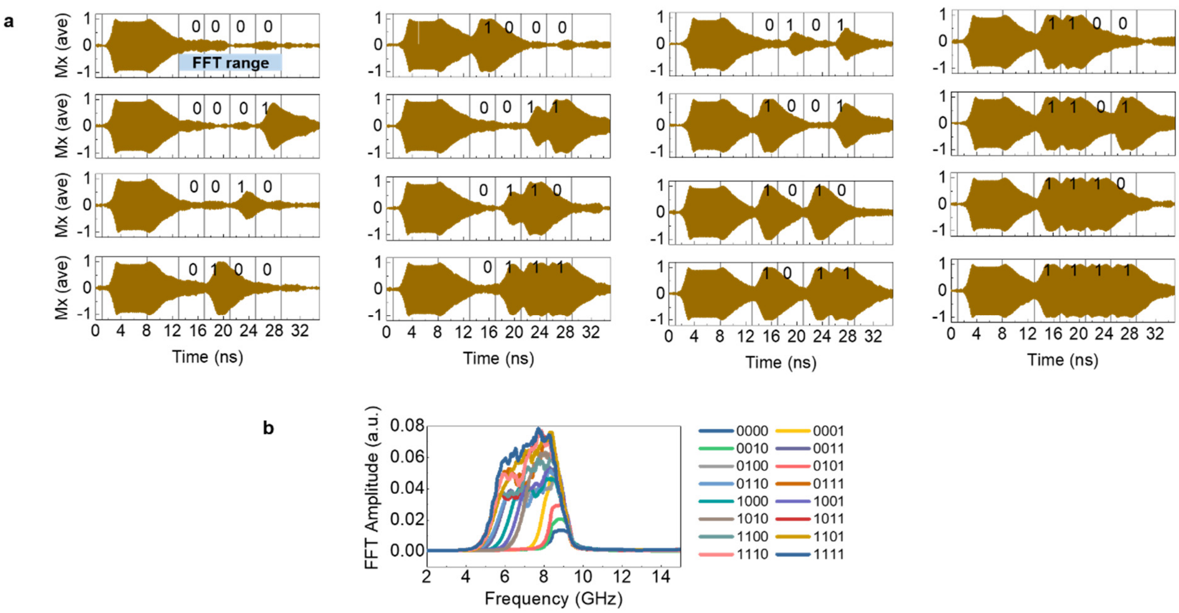

Figure. S8. Classification of inputs in the presence of thermal effects. Figure **a** shows the magnetization dynamics corresponding to the 16 different 4-bit input patterns for the modified pulse scheme with input parameters $I_e = 3.0$ mA, $\delta = 5$ ns, $I_0 = 1.2$ mA, $I_1 = 2.4$ mA, $\Delta t = 4$ ns, $\tau = 3.0$ ns at 300 K. Figure **b** displays the FFT amplitude spectra corresponding to the 16 different 4-bit input patterns. The current amplitude parameter $I_e = 3.0$ mA gives raise to auto-oscillatory mode at 300 K. However, the relaxation and classification property of SHO is not affected even in the presence of constant small amplitude oscillations driven by thermal effects.
